# Supplementary material for: Prevalence and associated factors of suicidal ideation and attempt among undergraduate medical students of Haramaya University, Ethiopia. A cross sectional study
Source: PLoS One. 2020 Aug 12;15(8):e0236398. doi: 10.1371/journal.pone.0236398 (PMC7423400; doi:10.1371/journal.pone.0236398)
Supplement: Attachment — Submitted filename: Response to Reviwers.docx [file pone.0236398.s002.docx]

**Dear Reviewers,**

It is my great pleasure to respond to your crucial and constructive comments. It helped me to know how much the scientific writing needs attention, specificity and clarity. I learned much as a junior researcher. Herewith I have addressed all comments and suggestions accordingly. Thanks once again.

Author,

Henock Asfaw

 1. Please ensure that your manuscript meets PLOS ONE's style requirements, including those for file naming. The PLOS ONE style templates can be found at https://journals.plos.org/plosone/s/file?id=wjVg/PLOSOne_formatting_sample_main_body.pdf and <https://journals.plos.org/plosone/s/file?id=ba62/PLOSOne_formatting_sample_title_authors_affiliations.pdf>

**Response:** Thank you. I have corrected the manuscript according to the Plos one journal guideline.

2.  We noticed you have some minor occurrence of overlapping text with the following previous work, which needs to be addressed: https://doi.org/10.1016/j.jflm.2014.08.006

Response: Thank you. I have addressed it.

In your revision ensure you cite all your sources (including your own works), and quote or rephrase any duplicated text outside the methods section. Further consideration is dependent on these concerns being addressed.
 
**3.** Please provide additional details regarding participant consent. In the Methods section, please state why it was not possible to obtain written consent, how verbal consent was recorded and whether the ethics committee approved this consent procedure. If your study included minors, state whether you obtained consent from parents or guardians.

**Response:** Thank you**.** The ethical consideration included under the method section.

**Ethical consideration**

Ethical approval was obtained from a joint ethical review committee of the University of Gondar, College of medicine and health sciences and Amanuel mental specialized hospital. Formal letter was obtained from Amanuel mental specialized hospital and submitted to Haramaya University, college of health and medical science for administrative approval prior to the data collection. Informed written consent was obtained from the students prior to the data collection. Those who did not wish to take part could be allowed either to withdraw from study at any time they want.

4. PLOS requires an ORCID iD for the corresponding author in Editorial Manager on papers submitted after December 6th, 2016. Please ensure that you have an ORCID iD and that it is validated in Editorial Manager. To do this, go to ‘Update my Information’ (in the upper left-hand corner of the main menu), and click on the Fetch/Validate link next to the ORCID field. This will take you to the ORCID site and allow you to create a new iD or authenticate a pre-existing iD in Editorial Manager. Please see the following video for instructions on linking an ORCID iD to your Editorial Manager account: <https://www.youtube.com/watch?v=_xcclfuvtxQ>

Response: Thank you. I have secured the ORCID ID.

5. Please amend either the abstract on the online submission form (via Edit Submission) or the abstract in the manuscript so that they are identical.

**Response:** Thank you. I will consider it and make it identical.

6. Please ensure that you refer to Figure 1 in your text as, if accepted, production will need this reference to link the reader to the figure.

**Response:** Thank you. Figure 1 included as reference under sample size and sampling technique section. It is provided as a separate file.

7. Please include your tables as part of your main manuscript and remove the individual files. Please note that supplementary tables (should remain/ be uploaded) as separate "supporting information" files.

**Response**: thank you. I have incorporated all tables in the manuscript.
Additional Editor Comments (if provided):

[Note: HTML markup is below. Please do not edit.]

Reviewers' comments:

Reviewer's Responses to Questions

Comments to the Author

1. Is the manuscript technically sound, and do the data support the conclusions?

The manuscript must describe a technically sound piece of scientific research with data that supports the conclusions. Experiments must have been conducted rigorously, with appropriate controls, replication, and sample sizes. The conclusions must be drawn appropriately based on the data presented.

Reviewer #1: Yes

Reviewer #2: Partly

2. Has the statistical analysis been performed appropriately and rigorously?

Reviewer #1: Yes

Reviewer #2: Yes

3. Have the authors made all data underlying the findings in their manuscript fully available?

The [PLOS Data policy](http://www.plosone.org/static/policies.action#sharing) requires authors to make all data underlying the findings described in their manuscript fully available without restriction, with rare exception (please refer to the Data Availability Statement in the manuscript PDF file). The data should be provided as part of the manuscript or its supporting information, or deposited to a public repository. For example, in addition to summary statistics, the data points behind means, medians and variance measures should be available. If there are restrictions on publicly sharing data—e.g. participant privacy or use of data from a third party—those must be specified.

Reviewer #1: Yes

Reviewer #2: Yes
4. Is the manuscript presented in an intelligible fashion and written in Standard English?
PLOS ONE does not copyedit accepted manuscripts, so the language in submitted articles must be clear, correct, and unambiguous. Any typographical or grammatical errors should be corrected at revision, so please note any specific errors here.

Reviewer #1: Yes
Reviewer #2: Yes

5. Review Comments to the Author

Please use the space provided to explain your answers to the questions above. You may also include additional comments for the author, including concerns about dual publication, research ethics, or publication ethics. (Please upload your review as an attachment if it exceeds 20,000 characters)

**Reviewer #1:**

**Comment:** This is, in summary, an interesting paper aimed to assess the prevalence and associated factors of suicidal ideations and attempts among undergraduate medical students of Haramaya University in a sample of 757 subjects. The study showed that the prevalence of suicidal ideation and attempts were 23.7% and 3.9%, respectively. Cumulative Grade Point Average, current alcohol use, depression, anxiety, and poor social support were significantly associated with suicidal ideation. In addition, depression and anxiety were linked to suicidal attempts.
The authors may find as follows my main comments/suggestions.
First, when within the Introduction section, the authors correctly reported that suicidal behavior is a major health priority being the second leading cause of death among adolescents and young adults, they might even briefly refer to the possible link between suicidal behavior, biological abnormalities, and emotional turmoil related to bereavement. Specifically, the emotional turmoil in suicide survivors of patients died by suicide may last a long time, and in some cases, may end with their own suicide. Thus, it is fundamental to understand the bereavement process after the suicide of a significant other in order to provide a proper care, and improve the outcomes. In order to briefly discuss this topic (although I understand that the link between suicidal behavior, and emotional turmoil related to bereavement process is not the main topic of the present manuscript), I suggest to discuss and cite, within the main text, the following papers (PMID: 31647957, 24082246, and 30601750).

In addition, specific biological abnormalities (e.g., prolactin, thyroid hormones, and immune alterations) may be significantly associated with suicidal behavior even involved in a complex compensatory mechanism to correct the abnormal central serotonin activity. The assumption that specific biological abnormalities may be associated or even predict suicidal behavior is of great importance, given the availability of such data in everyday clinical practice. Physicians of any kind as well as mental health professionals should be aware of the importance to insert as much information possible in the assessment of suicide. Thus, given the above information, my additional suggestion is also to briefly discuss and include throughout the manuscript the following additional reports (PMID: 30601750, 22748186, 29926090).
Moreover, as the main aims/objectives of this paper have been well described, the most relevant hypotheses underlying the present study should be reported as well in a detailed manner.
Furthermore, the section results is, in my opinion, divided in too many subsections that are difficult to follow for the general readership. Here, the most relevant study findings need to be better summarized for clarity.
In addition, the section limitations/shortcomings needs to be more directly updated as the most relevant caveats have been only partially described.
Also, the manuscript needs to be reviewed by a native English speaker for the quality of language.
Finally, what is the take-home message of this study? While the authors reported that the university should give due emphasis to reduce the risk factors and provide psychosocial support, they failed to report the most relevant conclusive remarks of their paper. Here, more details/information are required.
**Response:** Thank you. It is great recommendation to include the biological abnormalities related to suicidal behavior. The recommended articles helped me to get a better insight. I have incorporated briefly about the prolactin and thyroid hormone alterations linked to suicidal behavior. Introduction section. Para 7, line VII

I have considered the take- home message and have modified the conclusion part as follows”…. Cumulative grade point average, current alcohol use, depression, anxiety and poor social support were statistically associated with suicidal ideation, whereas Depression and anxiety were factors associated with suicidal attempts.” Para 1, line 3 to 5
**Reviewer #2:**

This is an interesting study with a potential clinical relevance focusing on suicidal behavior in undergraduate medical students. The paper present an important topic, with a concise introduction, adequate and well described methodology, well presented results and conclusions that are supported by the findings. A few comments to improve the paper:
**1.** The language of the paper should be tuned to be a bit more "scientific" please work on this. Also correct several typing mistakes, starting with capitalization of affiliation, missing or unnecessary spaces, etc.

**Response:** Thank you. The language have edited by native English speaker.
2. Abstract: the same applies. I believe also that if you provide a definition of suicide as PLOS has a general audience please keep it less "naive". Also provide more sound rationale in the abstract for this study. Also: conclusion of abstract should express more than prevalence was "high". Compared to what?

**Response:** Thank you. The first line of abstract section have modified. Para I. line 1.

Under the conclusion of abstract, I have re-written the conclusion by incorporating the study findings precisely. **Conclusion**: Para I, line 2 to 5.
3. **Methods:** there was a one-month data collection period. Suicidal behavior is linked to several stress- and environment related factors. Why I am not familiar with possible year-round fluctuations of Ethiopian weather, which is present in more northern home countries, the lack of such influencing factors should also be mentioned. Furthermore, I wonder how this period related to other possible risk stress factors like onset exam period. Therefore please comment on the choice of this period for data collection and the possible associated factors which may have to be considered when interpreting the data.

**Response:** Thank you. I do share your concern about the effect of stress and environment related factor on suicidal ideation and attempt. The weather in Ethiopia during May and June is the time with very favorable weather condition. The May is the spring season with occasional rain and hot weather while the **June** is summer season with a rain falls. So, by considering the weather situation we have collected the data from May 13 to June 12, which was the most comfortable and ideal with moderate hot and slight rainy weather. Concerning the exam, the data collection time was conducted during the exam free time (mid exam and final exam). The study participants were attending the regular class at that time.
4. Was the sample representative with respect to factors other than year of medical university? Such as socioeconomic background, family background, age, religion, etc?

**Response:** Thank you. The reason behind taking the students’ year of medical university was by assuming the other characteristics as normally distributed among classes. The participants were selected for the study by using simple random sampling method, so it gave them equal opportunity to be the part of study. After the random selection the subjects were interviewed for characteristics such as socioeconomic background, family background, age, religion etc.
5. How was suicidal ideation specifically assessed, by what question?

**Response:** Thank you. Suicidal ideation were assessed by “Yes” or “No” question coded as 1 for “Yes” and 0 for “No”. The items were adapted from module of world mental health survey initiative version of the WHO, CIDI (composite international diagnostic interview), in which suicide is studied and validated in Ethiopia both at clinical and community settings. Its internal consistence (cronbach alpha) in current study is 0.85. Data collection tool and procedure section. Data collection tool and procedure, Para III, line 1 to 4.
6. Was there correction for possible confounding variables?

**Response:** Thank you. As it is known the major aim of epidemiological studies are to identify risk factors of disease based on association. There will be also another factor associated with the exposure of disease and distort the exposure outcome relationship. We call such factors a confounding factor or variable. Such variable could be managed at study design phase and data analysis phase. So, the effect of confounding variable could be excluded from the final result. At the design phase we can use randomization, restriction and matching technique. At the analysis phase we can use stratification and multivariate models.

In current study we have used randomization technique. The study participants were selected randomly. Additionally during the analysis multivariate with logistic regression (bivariate and multivariate) analysis was conducted.
7. Were students with psychiatric disorders excluded? Was there screening for depression or anxiety and correction for these variables? Is there data on medications taken that could influence depression and suicidality in any direction?

**Response:** Thank you. Unfortunately there was no student with known psychiatric disorder among the study subjects according to the data we have got from the school of medicine head. Concerning the depression or anxiety, we have included the questions to screen them. Depression, anxiety and stress was collected by DASS, which have twenty one items and seven items each and used to assess emotional state of depression, anxiety and stress.

**Data collection tool and procedure,** Para I, line 6 to 9.

The information related to medication/substance use also included in our tool. ASSIST (Alcohol, Khat, Cigarette and Others) were used to collect data related to psychoactive substances which were developed by WHO. **Data collection tool and procedure,** Para I, line 4 to 5.
8. Is there data available in suicidal behavior prevalence in Ethiopia? It would be necessary to compare the prevalence found in this study to genera population data.

**Response:** Thank you. I have incorporated the prevalence of suicidal behavior among general population in Ethiopia. **Introduction.** Para VI, line 4 to 5.
In general although I have a few problems with the present study, the findings are interesting and the discussion is, while fully supported by the data, complex and thorough. After major revision the paper is worthy of publication.

6. PLOS authors have the option to publish the peer review history of their article ([what does this mean?](https://journals.plos.org/plosone/s/editorial-and-peer-review-process#loc-peer-review-history)). If published, this will include your full peer review and any attached files.
**Response:** Thank you. Am grateful to publish my future works on PloS one.
